# Factors associated with the permanence of doctoral students. A scoping review

**DOI:** 10.3389/fpsyg.2024.1390784

**Published:** 2024-10-28

**Authors:** Edna Hurtado, Esther Rosado, Martin Aoiz, Soledad Quero, Elkin O. Luis

**Affiliations:** ^1^Department of Psychology, School of Education and Psychology, University of Navarra, Pamplona, Spain; ^2^Instituto de La Familia, Universidad de La Sabana, Chía, Colombia; ^3^Institute of Modern Languages, University of Navarra, Pamplona, Spain; ^4^Universitat Jaume I, Castellón de la Plana, Spain; ^5^CIBER de Fisiopatología de la Obesidad y Nutrición (CIBEROBN), Madrid, Spain

**Keywords:** doctoral attrition, doctoral persistence, doctoral dropout, student affairs, doctoral education, higher education

## Abstract

The permanence of students in doctoral programs is a multi-causal phenomenon, which makes it difficult to address and leads to an isolated investigation of its causes, being necessary a joint understanding. The aim is to investigate, through a scoping review of quantitative studies published between 2015 and 2021, the influence of individual, academic, socioeconomic and institutional factors on retention, attrition and dropout. The 32 included studies evidenced a predominance of research focused on individual factors versus few that combined all possibly relevant factors. The present study provides evidence for the emergence of several subfactors: female students, self-efficacy and motivation (individual); the relationship with the supervisor (academic); support for psychological needs (institutional); and migratory status (socioeconomic). This article highlights the need for research that understands this problem with a multifactor approach and an impact on permanence.

## Introduction

The study of the permanence of students in their doctoral programs is relevant, not only because doctoral training is considered the most demanding process of teaching and learning and the culminating point of educational achievement (Jairam and Kahl, 2012), but also because it affects the university institutions (Skopek et al., 2022), as well as the generation of knowledge and the development of innovation in society. In this respect, the dropout of doctoral students implies high costs at the personal, institutional and societal levels.

Permanence is considered a multifaceted condition and has been defined from different perspectives. For example, Swail (2004) defined permanence as the ability of a student to remain in a university, whereas Berger and Lyon (2005), cited in Demetriou and Schmitz-Sciborski (2011), conceptualized permanence as “the desire and action of a student to stay within the system of higher education from beginning year through degree completion” (p. 12).

These two attempts to offer a definition of permanence reveal the effort made by authors to differentiate between the concepts of permanence and retention of students. In this respect, Serra (2010) states that permanence is oriented to the variables associated with the student, while retention is directly related to institutional capacities or variables, to guarantee the permanence and graduation of its students. On the other hand, Gómez Mendoza and Alzate Piedrahíta (2018) concluded that most of the studies analyzed refer to the permanence of students as the situation in which they finish and obtain their degree, and emphasized that the student who persists at the doctoral level is the one who does not interrupt his or her study plan. Furthermore, Tinto (1993) understands the permanence of the doctoral student as the sustained personal and intellectual interactions that occur within and between students and teachers and the various communities that make up the academic and social systems of the institution. Tinto’s definition encompasses both academic integration, defined as the relationships between students and faculty within a given academic field, and social integration, which encompasses the relationships between students and faculty outside the academic context.

Moreover, different models have been proposed to account for the factors that influence the permanence/dropout of university students. A first model proposes how conditions outside the university, such as psychological variables like sense of usefulness, satisfaction and stress, influence the decision to abandon a university degree (Bean and Metzner, 1996), whereas the model proposed by Spady (1970) considers that dropout is directly related to the student’s integration into the university environment.

A third conceptual model on the permanence/dropout of university students incorporates the theoretical developments of the student permanence model proposed by Tinto (1987) and the academic integration model of Bean and Metzner (1996). This third theoretical framework explains how student dropout is also a consequence of the student’s economic conditions. Finally, a fourth model, more focused on permanence, proposes that, in order to persist, students need to be part of formal academic systems and commit to the academic demands of their institutions. In addition, they need to participate in informal academic systems (relationships with faculty and administrative staff) and integrate into formal social systems by participating in the institutional activities outside their program of study that occur in the relationship with their peers (Tinto, 1993). This model of academic integration and social integration (Tinto, 1975, 1993) has been considered the most tested, cited, and respected approach to integration and retention (Simpson, 2003), and it will the one employed as the guiding framework for the present scoping review.

### Individual factors

In order to understand the determinants of dropout, Castaño et al. (2004) propose a classification based on four determinants. The first group presents the following descriptors: students’ age, gender and marital status, their family context, possible calamities and health problems, their social integration, time incompatibility with extracurricular activities, and unmet expectations. Authors such as Spady (1970), Tinto (1975), or Giovagnoli (2002) are associated with this first group of dropout determinants.

### Academic factors

The second group includes academic determinants, associated with the postulates of Spady (1970) and Tinto (1975), which include professional orientation, type of school, academic performance, program quality, study methods, student’s results in the entrance exam, their dissatisfaction with the program or other academic factors, and the number of subjects they have to take in their programs.

### Institutional factors

The third classification groups the institutional factors described mainly by Porto and Di Gresia (2000). These include academic normality, availability of scholarships and forms of financing, university resources, law enforcement, political environment, and the level of personal integration with teachers and students.

### Socioeconomic factors

The fourth and last category refers to socioeconomic determinants, studied in the research carried out by Tinto (1975) or Porto and Di Gresia (2000), among others. These include factors such as the socioeconomic stratum from which the student comes, his or her employment situation, economic dependence, whether they have dependents, their parents’ level of education, their parents’ employment status and income, and the macroeconomic environment.

### Results of previous reviews

Recent studies referring to individual aspects of doctoral students have found that these individuals show high levels of stress (Vekkaila et al., 2018), mental health problems such as depression (Byrom et al., 2022), or a deterioration in their physical health (Juniper et al., 2012). Despite the fact that these students have such negative perceptions about their physical or mental health, such difficulties have been commonly addressed and accepted as inherent to the training process (Byrom et al., 2022).

It has also been highlighted how individual and contextual attributes of the student influence their experience (Bard et al., 2000). Some studies highlight the importance of social interactions for the maintenance of motivation, e.g., modeling and teacher support, as determinants for the training of researchers (Pyhältö et al., 2020).

Other studies highlight the importance of formal and informal encounters that favor peer and faculty relationships (Hanson et al., 2020). Previous research has concluded that training in doctoral programs does not always provide a suitable environment to foster students’ motivation or well-being (Levecque et al., 2017).

High doctoral attrition rates can pose a financial and reputational challenge for universities, as a consequence of the direct relationship between research output and the work of doctoral students (Horta et al., 2018), in addition to the ordinary costs that doctoral programs have for institutions (Bair and Haworth, 2004). In this regard, authors such as Jaksztat et al. (2021) state that a high attrition rate implies an inefficient use of university facilities, which can jeopardize the success of the research carried out in such institutions.

### Research question

Following the recommendations of previous studies on the need to explore the relationships of factors associated with dropout as a whole rather than independently (Sverdlik et al., 2018), the present scoping review poses the following research question: What individual, socioeconomic, academic and institutional factors (phenomenon of interest) influence the permanence, attrition and dropout of doctoral students according to peer-reviewed investigations published between 2015 and 2021 in journals registered in SCOPUS AND Web of Science?

## Methods

The present scoping review was developed in accordance with the recommendations contained in the PRISMA Extension for Scoping Reviews (PRISMA – DcR): Checklist and Explanation (PRISMA; Tricco et al., 2018) and the description offered by Grant and Booth (2009; p. 101) as a type of review that provides “preliminary assessment of potential size and scope of available research literature.”

### Eligibility criteria

In the present review, the following publications were eligible: (1) refereed; (2) registered between January 2015 and September 2021; (3) in English or Spanish; (4) with keywords in title and/or abstract; and (5) indexed in both Scopus and Web of Science.

For the selection of the time window in which the articles were published, we took as reference the SCOPUS report downloaded in October 2021, which showed that the highest concentration of articles published on doctoral students was between January 2015 and September 2021, with a total of 4,440 different publications. Likewise, English and Spanish were chosen as the two publication languages of the articles to be included in the present investigation, as they were the two most used languages, accounting for more than 96% of the total (4,249 articles in English and 43 in Spanish).

To be included in the analyses, studies had to report on students in academic doctoral programs, employ the classification of the determinants associated with dropout described by Castaño et al. (2004), and have included individual variables such as age, support network, motivation, psychological needs, physical or mental health, gender, race, family, disability, or marital status. Furthermore, the present investigation included studies that had investigated academic variables such as academic trajectory, type of program, academic performance, learning strategies, admission processes, or academic load. With respect to the institutional studies were included when they had investigated variable such as academic normality, scholarships and forms of financing, university resources, integration with teachers and students, supervisors, or online programs. Studies were also selected for further analyses if they had focused on socioeconomic variables such as income, employment, or dependents (Intervention). Finally, studies that discussed doctoral student permanence, attrition, or dropout (Outcome) were also included in the sample.

Studies that reported on students in academic programs other than doctoral programs (high school, undergraduate, master’s, postdoctoral), or that had as their main focus supervisors, program directors, or administrative personnel (P) were excluded. As for intervention (I), studies were excluded from further analyses when they were related to curricular and efficiency models in education, internship and research policies, marketing strategies, the quality of academic programs, curricula for training in writing for research, or the use of information and communication technologies (ICTs). Furthermore, with regard to the results of the studies, those that did not report on the permanence, attrition or desertion of doctoral students were excluded. Finally, all studies with qualitative and mixed designs were excluded because of the objectives and expected results in the present study aim at collecting the available quantitative evidence so studies with such designs might not contribute to the strength of the evidence in a relevant way. Table 1 shows both the inclusion and exclusion criteria used in the present investigation.

**Table 1:** Inclusion and exclusion criteria.

Inclusion criteria	Exclusion criteria
1. Studies that include individual variables: age, support network, motivation, psychological needs, physical and mental health, gender, race, family, disability and marital status. 2. Studies that include academic variables: academic trajectory, type of program, academic performance, learning strategies, admissions processes, academic load. 3. Studies that include institutional variables: academic normality, scholarships and forms of financing, university resources, integration with professors, students and supervisors. Online programs. 4. Studies that include socioeconomic variables: income, employment, or dependents. Studies that assess retention, attrition or dropout of doctoral students. 5. Studies published in English and Spanish 6. Studies published between 2015 and 2021	1. Studies with student populations in academic programs other than doctoral programs (high school, undergraduate, master’s, postdoctoral). 2. Studies that have as their main target supervisors, program directors, or administrative personnel. Studies related to curricular and educational efficiency models, internship and research policies, marketing strategies, the quality of academic programs, curricula for research writing training, or the use of information and communication technologies (ICTs). 3. Studies that did not report on the retention, attrition or dropout of doctoral students. 4. In terms of research methodology, all studies with qualitative and mixed designs were excluded.

### Sources of information

The search was conducted in the meta-search engines UNIKA (Library at the University of Navarra) and EUREKA (Library at the University of La Sabana), and in the databases of Scopus and Web of Science. The references of the relevant articles retrieved were also examined to find additional studies using the snowball methodology (Berndt, 2020).

### Search strategy

In accordance with the use of the SPIDER methodology (Sánchez-Martín et al., 2023), from which the research question guiding this scoping review was formulated, the choice and organization of keywords for the search of the studies was made. Table 2 shows the elements employed within the SPIDER methodology, a strategy that allows the researcher to explore and synthetize information in such a broad topic as this.

**Table 2:** Elements employed with the SPIDER methodology.

SPIDER	Elements
I. Sample (S)	Students in doctoral programs
II. Phenomenon of interest (PI)	Studies that assess personal, academic, socioeconomic and institutional factors in relation to permanence, desertion and doctoral attrition, as described by Castaño et al. (2004).
III. Design (D)	Literature published in scientific journals, from quantitative studies
IV. Evaluation	Doctoral attrition, Doctoral persistence, Doctoral dropout, Desgaste doctoral, Persistencia doctoral, Deserción doctoral
V. Research type	Peer-reviewed quantitative studies published in journals indexed in SCOPUS and Web of Science databases January 2015 to December 2021.

Table 3 shows the keywords and Boolean terms used in the search.

**Table 3:** Keywords and Boolean terms employed in the search.

Keywords	Boolean	Keywords	Boolean	Keywords	Boolean
PhD students	AND	Self determination theory	AND	Doctoral attrition	OR
PhD students	AND	Perceived competence	AND	Doctoral persistence	OR
PhD students	AND	Academic motivation	AND	Doctoral dropout	
PhD students	AND	Making decisions			
PhD students	AND	Mental Health			
PhD students	AND	Student affairs			
Estudiantes de doctorado	AND	Teoría de la autodeterminación	AND	Desgaste doctoral	OR
Estudiantes de doctorado	AND	Competencia percibida	AND	Persistencia doctoral	OR
Estudiantes de doctorado	AND	Motivación académica	AND	Deserción doctoral	
Estudiantes de doctorado	YAND	Toma de decisiones			
Estudiantes de doctorado	YAND	Salud mental			
Estudiantes de doctorado	AND	Asuntos estudiantiles			

### Selection of studies

For the selection of the studies, the procedure defined by the PRISMA methodology was used. For the first identification stage, after 44 searches derived from keywords and Boolean terms, 899 studies were retrieved, distributed as follows: 286 in EUREKA, 328 in UNIKA, 67 in Scopus, and 218 in Web of Science. These searches and their corresponding results were coded and systematized in matrices. These matrices were entered into a spreadsheet to facilitate the organization of the information of each of the studies, by disaggregating into columns all the aspects that would later be analyzed in the different phases of the screening.

Next, a careful review was made of: (1) title, (2) year of publication, and (3) abstract, to determine if the initially selected studies meet the inclusion criteria or had to be dismissed because of the exclusion criteria. For those articles whose abstract did not present sufficient evidence to be placed in one of the inclusion or exclusion categories, a third grouping called “undefined” was created with the aim of classifying them, at a later stage, based on a complete reading and on a more in-depth review of each of these studies. Additionally, the quality of the articles was assessed using the EACSH (López-López et al., 2019), a scale to determine the quality of scientific articles in social and human sciences, and 96% of the studies showed ratings between a very high and medium-high level.

### Data extraction

After this preliminary classification of the studies, the researchers created a data extraction matrix that included the following elements: (1) APA-type citation, (2) first author, (3) country of publication, (4) country in which the study was conducted, (5) type of study, (6) type of data collection (cross-sectional or longitudinal), (7) type of sampling, (8) participant definition, (9) sample size of participants, (10) mean age, (11) standard deviation of age, (12) percentage of female participation, (13) social status, (14) race, (15) purpose of the study, (16) dependent variables, (17) independent variables, (18) research question, (19) research instruments, and (20) results oriented to the description and relationship of the factors associated with the permanence, attrition and dropout of students in doctoral programs.

Two of the investigators independently read and evaluated each of the studies included in the sample. The entire team then analyzed the overlaps as well as the divergences in the extractions made by those two investigators. If there was a divergence on any category of analysis, the two initial investigators discussed the cause: if the cause was an error in the analysis, it was corrected; when the divergence was due to a difference in the origin of the data supporting the finding, it was decided whether or not to include that information.

### Data analysis

After analyzing the data with a quantitative description of the characteristics of the studies collected in the extraction matrix, a qualitative analysis was initiated using the web version of the ATLAS.ti software (Version 3.15.0–2022-03-09). In this analysis, a thematic exploration was carried out based on the elaboration of a codebook, built on the following deductive categories of analysis (Castaño et al., 2004): (1) Individual factors: age, gender, marital status, family context, calamity and health problems, social integration, time incompatibility with extracurricular activities, unmet expectations; (2) Academic factors: professional orientation, type of institution, academic performance, quality of the program, study methods, entrance exam results, dissatisfaction with the program and other academic factors, number of subjects; (3) Institutional factors: academic normality, scholarships and forms of financing, university resources, law enforcement, political environment, level of personal interaction with teachers and students; and (4) Socioeconomic factors: socioeconomic stratum, employment status, parents’ employment status and income, economic dependence, dependents, parents’ educational level, macroeconomic context of the country. Additionally, inductive (emergent) categories of analysis were extracted, and the codes of attrition, permanence and dropout were included, according to the evaluation guidelines in the SPIDER tool (Methley et al., 2014). This way of proceeding resulted in the configuration of a deductive approach whereby, by looking at the frequency of the codes in the studies, an analysis of the relationship between them and of co-occurrences was made, based on the central model stated in the codebook. In a final stage, the thematic networks were constructed on the basis of the central model, and then entered into the analysis of co-occurrences.

## Results

### Selection of studies

**Figure 1:**
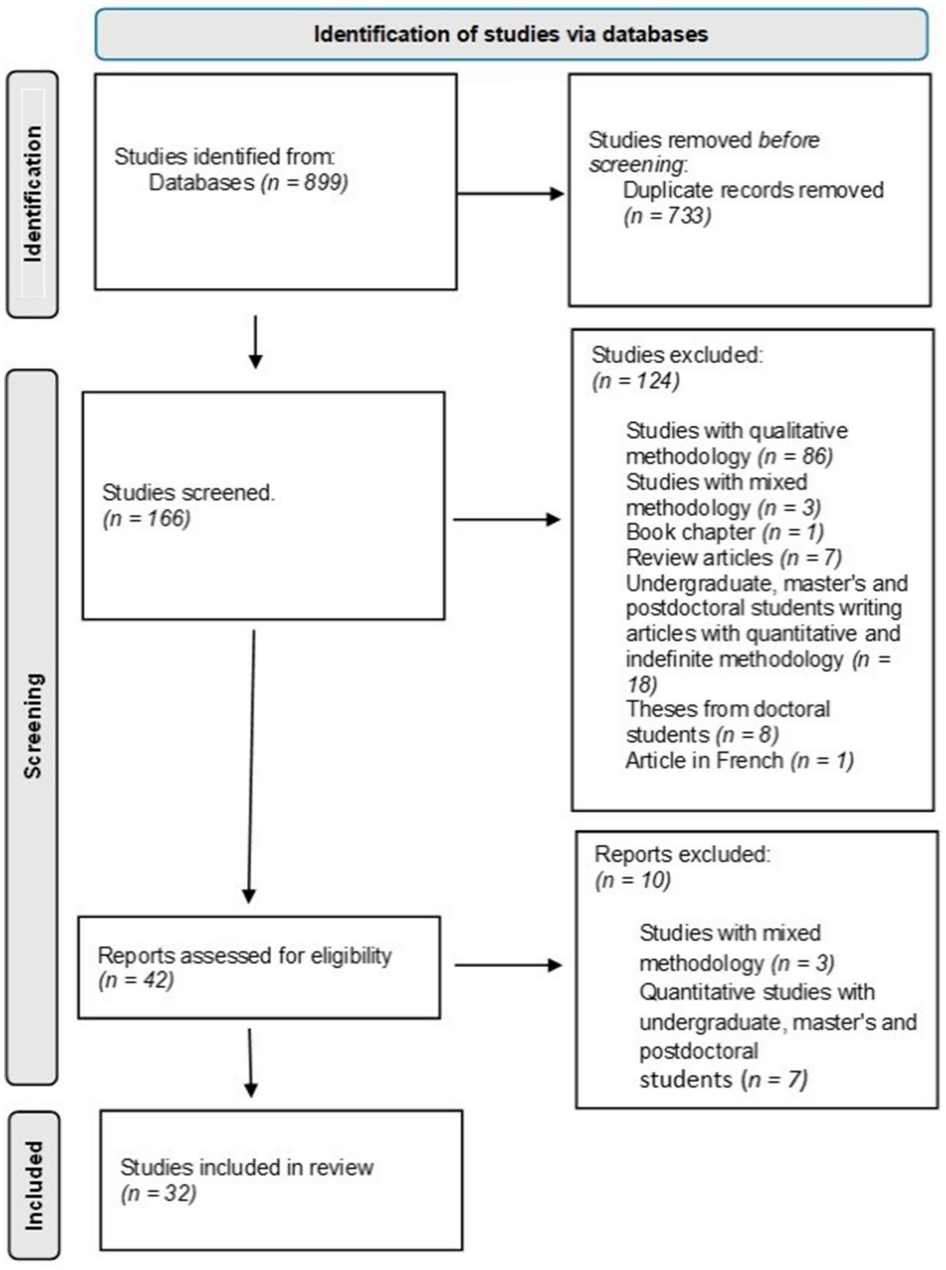
Flow diagram of the procedure followed in the selection of studies according to the PRISMA methodology.

From an initial selection of 899 articles, after eliminating duplicate studies and applying the exclusion criteria, the sample consisted of 32 studies (Figure 1).

### Descriptive analysis of the studies included in the final analysis

Half of the studies analyzed in the present investigation (16) had been conducted at universities located in the United States, while studies from Canada (*n* = 3) and Belgium (*n* = 3) each accounted for 9.4% of the analyzed sample (18.75% in total). Regarding the year of publication of the studies, a progressive increase in the number of studies published is evident, from one study in 2015 to 5 studies in 2021. The highest number of studies is recorded in 2020 with 9 studies.

With respect to the information about the population that some of the studies reported, it was observed that their sample sizes ranged from 18 to 3,004 doctoral students (average *N* = 914), where 62% of the participants were women. Likewise, the mean age of the participants was 32.2 years, ranging from 18 to 80 years. Detailed information about the populations in the studies included in this investigation is presented in Supplementary Table S1.

According to the type of methodology employed in these studies, most were cross-sectional (78.1%), with either an observational (42%) or descriptive (58%) approach, i.e., none of the 32 investigations included in our analysis used an experimental approach. With respect to the manner in which their samples were selected, the vast majority of the studies (90.6%) used non-probability convenience sampling.

On the other hand, the analysis of the measurement instruments reported in these studies enabled a first classification of information by factors associated with permanence or dropout. Of the 58 instruments used, 65.5% measured individual factors, 12.1% academic factors, 15.5% institutional factors, and 6.9% socioeconomic factors.

As for the limitations reported by the studies themselves, 50% of these studies mention the characteristics of their sample and their implications for the results of the study (De Clercq et al., 2021; Holmes and Rockinson-Szapkiw, 2020; Sverdlik and Hall, 2020). A second limitation was the inherent restriction in analyzing such educational processes because they are limited to studies in a given country, an issue already mentioned by Gruzdev et al. (2020). Furthermore, the studies carried out by Hands (2020) admit that the inclusion of samples of students from a single discipline limits the generalization of their findings. A summary of the main findings in each of these investigations, including the scoping review results can be found in Supplementary Table S2.

### Content analysis of the studies

For the analysis process, a codebook was developed containing both the factors associated with dropout (Castaño et al., 2004) and the previously mentioned subfactors. Once the codebook was defined, the thematic networks for each of the factors were constructed. For this analysis within the ATLAS.ti software, groundedness is equivalent to the number of citations in the studies to which a code or category is related (Justicia, 2005).

The thematic network of the individual factor is the one with the highest number in its groundedness (233). Within this network the following subfactors were found, organized in descending order according to the frequency of groundedness: family context, calamity and health problems, unmet expectations, social integration, age, gender, marital status, time incompatibility with extracurricular activities and satisfaction – success. The emerging categories presented a total of 6 subfactors, according to their frequency: motivation, self-determination, well-being, self-efficacy, women and exhaustion.

The next factor analyzed in relation to its thematic network was the academic one. The following subfactors, according to their groundedness, emerged here: type of educational institution or university, professional orientation, program quality, academic performance, results in the entrance exam, study methods, number of subjects, and dissatisfaction with the program and other academic factors. With respect to the academic factor, four subfactors also emerged that had not been detected in the analyses carried out by previous research: supervisor, appropriation of the research project, perceived progress, and online learning (in descending order of frequency).

The thematic network of the institutional factor and its groundedness consisted of the following subfactors: level of personal interaction with teachers and students, university resources, and scholarships and forms of financing. However, the subfactors of the political environment, academic normality and law enforcement did not show any groundedness. Within the analysis of the institutional factor, however, the institutional support for psychological needs was recorded as an emerging category.

**Figure 2:**
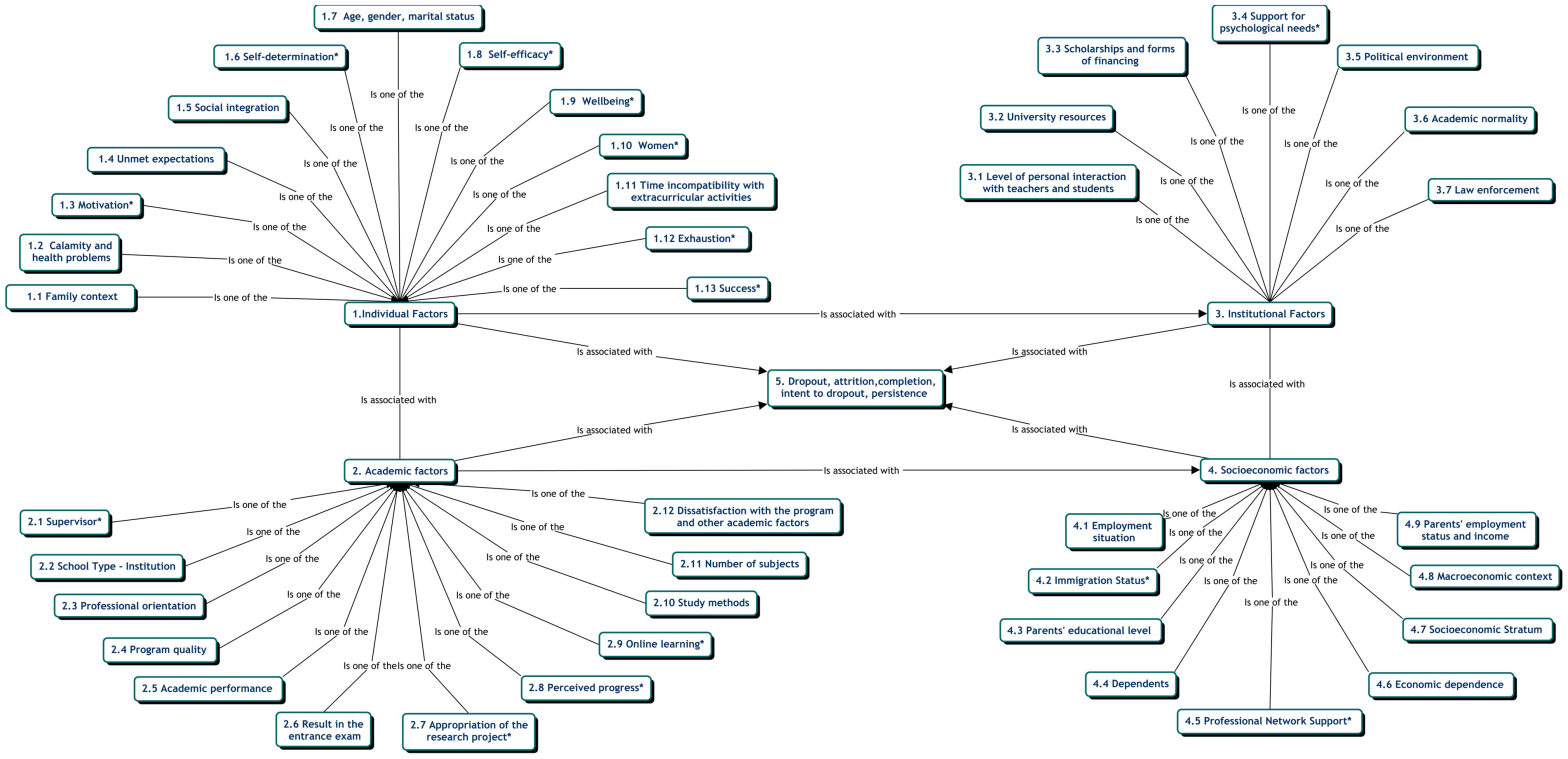
Thematic network of factors associated with dropout and emerging categories.

Finally, the analysis of the socioeconomic factor detected the following subfactors: employment situation, parents’ educational level, dependents, economic dependence, and socioeconomic stratum. The macroeconomic context and the parents’ employment status and income did not show any groundedness, but within the socioeconomic factor, the analyses found two emerging subfactors: immigration status and professional network support (Figure 2).

Once the data were analyzed independently, according to each of the four factors associated with dropout, they were related to their subfactors (Castaño et al., 2004). In addition, the categories emerging from this analysis were studied in relation to the following categories: dropout, attrition, completion and intent to drop out. Of the four thematic networks the individual factor, 233 quotations (groundedness) were found, with a higher number (66) for family context. In the studies analyzed in the present investigation, the groundedness for the academic factors was 120, with 43 quotations referring to the emergent supervisor subfactor. Institutional factors, in turn, presented 55 quotations, while socioeconomic factors showed a smaller groundedness with only 24 quotations. Both institutional and socioeconomic factors included an emergent subfactor: support for psychological needs and professional network support, respectively. It should be clarified that, in the same publication, researchers may have addressed one or more factors and subfactors.

To broaden the understanding of the influence of individual, academic, institutional and socioeconomic factors associated with student dropout in their doctoral programs, based on the construction of the thematic networks (factors, deductive and emergent subfactors), it was found that the presence of a factor or subfactor in each study did not exclude the rest, but on the contrary, all authors measured more than one factor or subfactor in their research.

### Co-occurrence analysis between factors

A co-occurrence analysis, i.e., a record of all related factors in the same study, was used to study those cases in which the researchers studied more than one factor or more than one subfactor. In this regard, the relationship between individual and academic factors presented the highest number of co-occurrences (22). Furthermore, the studies tended to relate academic factors such as professional orientation, supervisor, type of institution, academic performance and program quality, with the following individual factors: age, gender and marital status, self-efficacy, well-being, satisfaction – success, calamity and health problems, social integration, unmet expectations, motivation, exhaustion, self-determination and family context. The second highest frequency of co-occurrences was found between individual and institutional factors (11), with 5 co-occurrences between the level of social integration (individual) and the level of personal integration of teachers and students (institutional).

Within the co-occurrences between the individual and institutional factors, a relationship was also found between the individual subfactor of family context and the institutional subfactors of scholarships and forms of financing, level of personal interaction with teachers and students, and support for psychological needs. In addition, the subfactors of the individual factor social integration, motivation and family context were found to present co-occurrences with the institutional subfactor support for psychological needs. On the other hand, the institutional subfactor university resources presented co-occurrences with the individual subfactor well-being, a finding that is consistent with the frequency with which the word support appears in the studies.

Three co-occurrence analyses were performed: (1) between the individual factors and the academic factors and subfactors, (2) between the institutional factors and the socioeconomic factors and subfactors, and (3) between each of the subfactors that appeared within each factor. Regarding the relationships between the individual factors (well-being, calamity and health problems, social integration, motivation, women, and family context) and the socioeconomic factors (socioeconomic stratum, parents’ employment status and income, economic dependence, dependents, parents’ educational level, macroeconomic context of the country, immigration status, and professional network support), a decrease was observed in terms of frequency in the co-occurrences when compared to the results obtained in the co-occurrences of the individual factors with the academic and institutional factors. This decrease was even greater in the number of co-occurrences between the academic and institutional factors and subfactors.

On the other hand, in the relationships between the institutional and socioeconomic factors, co-occurrences were found between the institutional subfactor level of personal interaction with teachers and students and the socioeconomic subfactor work situation. Finally, between the academic and socioeconomic factors no relationship value was found, either at the global level or between the subfactors.

### Co-occurrence analysis with the categories: dropout, attrition, completion, intent to drop out and permanence

The presence in the studies of the analysis categories of dropout, attrition, completion, intent to drop out and permanence was also coded, as were the co-occurrences among them and with each of the factors at the general level. In this way, relationships could be established not only between the factors associated with dropout (Castaño et al., 2004), but also within the categories themselves, as well as between them and the aforementioned factors. These categories were included in the analysis because they were already part of the search words and appeared with a greater number of frequencies in the analysis of the 32 studies in the word cloud that was generated by the software ATLAS.ti, typical of this type of analysis.

The studies show the highest relationship between the factors associated with the dropout category, with a frequency of 44 co-occurrences, followed by permanence (34 co-occurrences), attrition (19 co-occurrences), intent to drop out (13 co-occurrences), and completion (12 co-occurrences). In this set of frequencies, the relationship is based on data taken either directly from students who are pursuing their doctoral studies, or from the academic records of students who pursued it at the time, but subsequently dropped out of the program (Table 4).

**Table 4:** Co-occurrence of factors in the categories of dropout, attrition, completion, intent to dropout, and persistence.

Factors	Dropout	Attrition	Completion	Intent to drop out	Persistence
Individual	18	5	4	8	16
Academic	16	8	5	2	9
Institutional	5	3	2	1	5
Socioeconomic	5	3	2	1	4

As in previous analyses, the highest number of co-occurrences is found in the relationships between individual factors and the categories analyzed, except in the category of completion, where academic factors co-occur more frequently.

### COVID-19 update

With the aim of considering in the present study a historically relevant event such as the COVID-19 pandemic, an additional search process was conducted in April 2024, with the same criteria employed as in 2022. Although it yielded a total of 43 articles, only 13 investigations matched those eligibility criteria and were subsequently analyzed in depth. The main conclusion to be drawn from such analysis is that the variables associated with the permanence of doctoral students most studied were the individual ones in terms of mental health and the institutional ones in terms of admission processes and the need for institutional support by the universities.

In particular, Paucsik et al. (2022) highlight an increase in depression, anxiety and stress, which implies a decrease in well-being. Similarly, Aristeidou and Aristidou (2023) also reported that three out of four doctoral students experienced depression mainly associated with the care of children living with them and the lack of funding.

On the other hand, Tu et al. (2023) concludes that experiencing a negative impact due to the pandemic and reflecting on such adverse event correlates with higher PTG (Post Traumatic Growth). Furthermore, they also evidence the need for institutional policies to manage risks and build resilience in academic communities. Likewise, Smith et al. (2024) underscore the relevance of having psychological structural supports in the institutions.

The research teams in those 13 studies show their concern for the mental health of doctoral students during the COVID-19 pandemic. However, only one of the articles explored the academic quality in relation to the increase in the admission of students to doctoral programs, during this time, with non-academic motivation and absence of academic skills, which questions the graduation of these students (Maloshonok et al., 2023).

As can be seen the studies published from September 2021 to May 2024 report variables that have already been assessed in the initial sample, which corroborates the reliability of the results obtained in the analysis of the studies in the initial sample of the present review.

## Discussion

This scoping review aimed to identify, from the literature published between 2015 and 2021, how individual, academic, institutional and socioeconomic factors (Castaño et al., 2004; Tinto, 1975), are related to doctoral student permanence, attrition and dropout. To the best of our knowledge, the present research constitutes the first review that attempts to identify the relationship between the aforementioned factors and the permanence of doctoral students.

This scoping review has shown the diverse attention that researchers have paid to the different factors: individual factors have the highest frequency (19%), followed by institutional (13%), socioeconomic (10%), and academic (9%). An interesting finding is the existence of studies that integrated the four factors (9%) and also of studies that focused on the combination of individual and academic factors (28%), individual and institutional (6%), individual and socioeconomic (3%) and academic and institutional factors (3%). In this regard, authors such as Jackman et al. (2022) point out how inadequate the study of factors in isolation can be and highlight the need to incorporate all of them in order to understand the doctoral experience holistically, in addition to helping to structure intervention plans that are better adjusted to the reality of doctoral students.

In this sense, there is a consensus on the need for research that encompasses the entire reality of the doctoral student and his or her different contexts, which can be reflected in the model of authors such as Tinto (1975, 1993), who proposes the need to study formal and informal academic systems, as well as the integration of people in social systems.

The results show the majority of studies focus on individual factors. This contrasts with the lower percentage (9%) of studies that combine all factors. These findings agree with Skopek et al.’s (2022) point of view (2022), who state that research on dropout at the doctoral level and on the time elapsed to obtain the corresponding degree has focused on the sociodemographic and individual characteristics of the students, so its scope is limited when it comes to addressing the complexity inherent in the doctoral training of students. Furthermore, the most recent studies analyzed (2022–2024), conducted in the midst of the COVID-19 pandemic, evidence the researchers’ concern for students’ mental health. This fact contributes to the increase in the number of studies that focus solely on individual factors leaving aside other factors such as academics, which may also affect student retention (Maloshonok et al., 2023).

### Doctoral permanence is a multifactorial issue

The exploration of the factors studied in the different research studies through the systematization of the information in the semantic networks used for this review enriched the factors proposed in both Tinto’s (1993) and Castaño et al.’s (2004) models, since, by means of this analysis, particular subfactors emerged as determinants of student retention.

#### Individual factors

In the case of individual factors, of the six emerging subfactors reported in the results section, the emerging factor women has been a finding that evidences interest on the part of researchers. This is likely because of the increased participation of women in doctoral programs (Volkert et al., 2018). Another possible explanation is reported by authors such as Epstein and Fischer (2017) when drawing attention to the high dropout rates of female science students compared to males, finding in self-efficacy (another emerging subfactor) an opportunity for intervention to increase the levels of permanence in doctoral programs. Furthermore, Epstein and Fischer (2017), concluded that women may postpone motherhood due to their doctoral work or even discard their future academic careers. Unfortunately, this study is the only one of all those analyzed that addresses this issue, so it might be interesting to conduct further research on this, including the impact that motherhood may have on the success of doctoral students.

Likewise, the emerging factor motivation is one of the most studied by researchers, since it is found in about 70% of the analyzed research, so we can observe a consensus among researchers on the relationship and the impact that the motivational profiles of students have on the permanence and completion of their doctoral studies (De Clercq et al., 2021; Sverdlik and Hall, 2020).

#### Academic factors

Within the academic factors, the three subfactors reported by the present study are characteristic of the doctoral training environment, as is evident from the studies analyzed in the present investigation. One aspect noted as important by several of the studies analyzed was the relationship with the supervisor. Authors such as Gruzdev et al. (2020) found among students a higher level of both satisfaction and compliance with regulatory deadlines (enrollment times, delivery of reports, estimated time for the degree), when they had supervisors with high levels of involvement in the doctoral work, and who were concerned about how their doctoral students adapted to the academic world. Litalien and Guay (2015) corroborate this by stating that the support of the supervisor, teachers and other academic staff improves the perceived competence of the student, which in turn generates a lower risk of dropping out of the program. In this relationship between individual factors and academic factors, it stands out that the perceived competence and the supervisor appear as emerging subfactors within the individual and academic factors, respectively.

#### Institutional factors

The only emerging subfactor within the institutional factors is support for psychological needs. In this regard Schwoerer et al. (2021) highlight the importance for doctoral students of sources of social, emotional, mentor (supervisor) and professional peer support. In fact, institutional support is considered as a determinant for the promotion of health (physical and mental) of doctoral students (Volkert et al., 2018). In addition, several studies show that greater support reduces stress levels, which influences the student’s perception of balance between their work and personal life (Ribau and Alves, 2018).

#### Socioeconomic factors

Another result of the present study was the relationship between the emerging socioeconomic subfactor migration status and the type of program (academic subfactor). This relationship is reflected in the lower success rate of non-European students, specifically, in social sciences (De Clercq et al., 2021). Similarly, this study identified that switching university frequently predicts higher dropout rates among European doctoral students.

Wollast et al. (2018) conducted an interesting analysis of individual, academic, institutional, and socioeconomic factors. These authors concluded that students who are single, with low master’s degree grades, without scholarship, belonging to the fields of social sciences and humanities and aged over 26 are 50% more likely to drop out of their doctoral program. Therefore, it is essential to recognize those factors or socioeconomic profiles of risk of dropout in this type of population, so that, in this way, institutions can facilitate actions to reduce their potential negative impact.

### Permanence vs. dropout of doctoral students

The present review has identified a marked interest of researchers to study the permanence of doctoral students. The vast majority of the studies included in the present analysis (87%) have focused on investigating the permanence rather than on attrition or dropout from doctoral programs. This may be due either to the availability of the target population, or to an interest of researchers and institutions to have information that helps to prevent situations that may lead to dropout in doctoral studies, and thus increase success rates in these programs (Gómez Mendoza and Alzate Piedrahíta, 2018), as opposed to those studies focused on dropout, where the possibility of some type of intervention with students is lost. However, it is necessary to clarify that the concept of permanence is based on the definitions of dropout and its rates reported by other researchers or by the institutional measurements performed.

In this regard, Rockinson-Szapkiw et al. (2016) analyzed institutional (financial support, program, curriculum, and support services) and integration (academic, social, economic, and family) variables with the objective of distinguishing those affecting permanence or dropout. The results showed that support services, program quality, curriculum, and academic integration (with faculty and family) were factors that helped to explain permanence.

### Relationships between factors to work on the permanence of doctoral students

The relationship between different individual, academic, institutional and socioeconomic factors has been addressed by authors such as Wollast et al. (2018), who studied the following variables: nationality, marital status, master’s degree, age at enrollment, field of research, permanence at the same university, and funding. In their research they found that the marital status (individual subfactor) is a predictor of the study completion (success) rate, since there is a higher relative percentage of married doctoral students who complete their doctoral programs. On the other hand, in relation to institutional factors, they identified that students with a research grant in health sciences have better permanence rates compared to students without a grant in humanities and social sciences programs, who show higher dropout rates.

Regarding the mental well-being and stress of doctoral students, Miller and Orsillo (2020) showed that both the acceptance of experiences and valued living act as protectors against depression, anxiety and stress. Along the same vein, Byrom et al. (2022) observed that the supervisor’s support (academic subfactor) and the perceived self-confidence (individual subfactor) decrease stress levels. Likewise, these authors found that self-confidence predicts the mental well-being of doctoral students, that family support correlates positively with achievement orientation, and that general health and hours of sleep decrease predicted stress levels and increase mental well-being. Schwoerer et al. (2021), in turn, showed that doctoral students who perceive greater support from their supervisor report less stress and fewer work-life conflicts. However, these same authors found that, when evaluating only the mediating role of academic support from the supervisor, this role does not have an effect on the decrease in the stress that the doctoral student perceives.

One aspect that the studies analyzed consider keeping in mind in terms of the perception of well-being is the experience of the impostor syndrome (Clance, 1985) that doctoral students might experience. In this regard, authors such as Sverdlik et al. (2020) concluded that doctoral students’ perceptions of their belonging to the academic community are positively related to lower levels of such syndrome. Tao and Gloria (2019) studied the moderating effect of gender (women) on the relationship between the impostor syndrome and the beliefs on academic permanence in STEM (Science – Technology – Engineering – Mathematics) programs, and highlighted that this relationship is stronger in programs in which there is a higher number of women, which was evidenced by low perceptions of academic self-efficacy and negative perceptions regarding the research and training environment.

Lee et al. (2020) concluded that the strongest positive predictor for permanence is the student-faculty relationship, above technological factors, knowledge, skills, self-efficacy, or intentions to persist. Other studies, such as the one conducted by Estrada et al. (2019), concluded that for historically underrepresented students, the student’s scientific identity significantly influences permanence by valuing it as a mediating variable between social support and the intention to persist. These results demonstrate the value of doctoral student integration and relate to the emerging subfactors of well-being (individual factors), supervisor (academic), psychological needs support (institutional), and professional network support (socioeconomic), which in turn supports the concept of integration put forth by Tinto (1993).

### Limitations

The present scoping review has only analyzed studies focused on the student, so in future research it would be valuable to integrate the information reported by either the universities or the supervisors themselves, in order to provide a more complete perspective that can account for various processes of mental health support at both individual and academic levels. Among others, this could include supervision, academic processes and scholarship systems, as well as the processes of employment linkage as employers.

Another limitation of the present study was the exclusion of studies that did not present a quantitative methodology. Future research should incorporate studies with mixed and qualitative methodology into the analysis, providing evidence on the perceptions of all the individuals involved in the development of a doctoral thesis.

Finally, only five longitudinal studies were found (16% of the total), which prevents us from having findings that allow a complete understanding of the student’s trajectory. Most of the researchers made cross-sectional measurements with the participation of students enrolled in doctoral programs, which prevents them from knowing the relationships of these students with dropout and focuses the results only on the characteristics associated with the permanence and retention of doctoral students at university.

## Conclusion

This literature review identified a wide range of relationships among individual, academic, institutional, and socioeconomic factors with respect to doctoral student permanence, attrition, and dropout.

The in-depth study of 32 publications, after a rigorous search and corresponding screening, enabled a two-level analysis. The first, descriptive, allowed us to evidence a growing interest in the topic of study, as reflected in the increase in publications since 2018, a greater number of studies reported in North America (50%), a high prevalence of cross-sectional studies (78%), and a greater number of studies focusing on the permanence of doctoral students (47%). Likewise, in order to understand each of the factors associated with the permanence of doctoral students, the identification of the 58 instruments used by researchers to measure these factors was of great importance.

The second analysis, carried out with the help of the software ATLAS.ti, made it possible to investigate the frequency and type of relationships between factors and subfactors that Castaño et al. (2004) had previously mentioned. Taking these categories as a starting point, an inductive analysis was performed, later complemented by a deductive analysis that provided 13 emerging categories specific to the population under study, thus enriching the initial approach of these authors. The report of the frequencies of the codes in terms of their groundedness made it possible to answer the question about the factors that researchers had studied the most (i.e., the individual ones). Likewise, the co-occurrence analyses enabled the study of the relationship between the different factors, also in terms of frequency, showing that the most studied relationship had been the one between individual and academic factors, while the least studied is the relationship between institutional and socioeconomic factors (Skopek et al., 2022).

The integration of the four factors associated with permanence in a single study is fundamental, as permanence is a multifactorial issue. However, according to our findings, only 9% of the studies assessed in this scoping review reported a broad integration of factors. This supports the need to continue working on developing research studies that integrate as many factors as possible. Likewise, the association of factors around student retention is relevant, as measuring and intervening with students during their academic trajectory could lead to better graduation rates and to a decrease in the number of doctoral students who drop out of their programs. In turn, this integration could also favor the participation of the different actors that play a direct or indirect role in the training process (supervisor, professors, colleagues, family, friends, administrative staff, etc.), allowing a comprehensive attention to the permanence of students.

According to the findings reported in the different studies, it can be concluded that both the prevention of desertion and the promotion of permanence are the result of comprehensive attention to the doctoral student. Isolated actions may not have the desired effects if it is not the university institution itself that offers a comprehensive solution that addresses the characteristics of each student. This solution should include fundamental aspects for academic training such as the relationship with the supervisor, the presence of help centers to assist the student population with respect to their migratory status or their need to access scholarships or financial aid, as well as strategies for the well-being of these students that include formal and informal support networks at different levels, from colleagues to friends and family.

This scoping review provides integrative results by going beyond a descriptive analysis of the studies. Furthermore, it offers in-depth findings on the factors that previous research has identified as having an impact on the permanence and dropout of doctoral students. These findings also encourage the need for future research that contributes to the comprehensive understanding of the process and to the timely accompaniment through the interaction of the different factors.

Finally, it is noteworthy that the studies analyzed within the additional literature review to include the possible influence of COVID-19 pandemic (January 2022–May 2024) show a primary interest in the study of the mental health of doctoral students. Although this is an individual subfactor that had been frequently addressed prior to the pandemic, most recent studies have added to this subfactor the pandemic-specific implications of situations such as confinement.

## Data Availability

The raw data supporting the conclusions of this article will be made available by the authors, without undue reservation.
