# Supplementary material for: Factors associated with the permanence of doctoral students. A scoping review
Source: Front Psychol. 2024 Oct 28;15:1390784. doi: 10.3389/fpsyg.2024.1390784 (PMC11550976; doi:10.3389/fpsyg.2024.1390784)
Supplement: Supplementary file 1 [file Data_Sheet_1.docx]

**Supplementary material**

**Table 1**

*Description of participants*

| **Author(s), year of publication and country** | ***N*** | **Age** | **Gender** | **Ethnicity** |
| --- | --- | --- | --- | --- |
| Alkathiri, M. S. (2019). United States. | 45 | 35 or younger: 33.3%.  36 or older: 66.7%. | Female: 68.9%  Male: 31.1% | Does not report |
| Ames, C., Berman, R., & Casteel, A. (2018). | 698 | Does not report | Does not report | Does not report |
| Bekova, S., & Dzhafarova, Z. (2019). Russia. | 2,020 | Does not report | Female: 45%  Male: 55% | Does not report |
| Boykin, T. F., & Walker, L. J. (2018). United States. | 238 | Does not report | Does not report | Does not report |
| Byrom, N. C., Dinu, L., Kirkman, A., & Hughes, G. (2022). United Kingdom. | 431 | 21-25 years = 33.6%.  26-30 years = 33.4%.  over 30 years = 32.9%. | Female: 67.7%.  Male: 32.2 | British White: 52.4%  Another White: 25.3%  BAMEB: 22.3% |
| De Clercq, M., Frenay, M., Azzi, A., Klein, O., & Galand, B. (2021). Belgium. | 461 | Average age: 28.2 | Female: 61.4%  Male: 38.6% | Does not report |
| Estrada, M., Zhi, Q., Nwankwo, E., & Gershon, R. (2019). United States. | 101 | Average age: 28 | Majority group:  - Female 70%  - Male 30%  HU Group  - Female 60%  - Male 40% | African-Americans: 48%.  American Indians: 12%.  Pacific Islanders: 8%.  Asians / Asian Americans: 20%.  White (disabled, biracial or self-identified): 12%. |
| Gasa, V., & Gumbo, M. (2021). Ethiopia. | 120 | 28 or younger: 25.7%.  29 or older: 74.3%. | Female: 3.9%  Male: 96.1% | Does not report |
| Gruzdev, I., Terentev, E., & Dzhafarova, Z. (2020). Russia. | 2,034 | Does not report | Female: 45%  Male: 55% | Does not report |
| Hands, A. S. (2020). United States, Canada. | 18 | 18-24 years = 5.5%.  25-34 years = 66.7%.  35-44 years = 11.1%.  45-54 years = 5.5%.  55-64 years = 11.1%. | Female: 50  Male: 50%. | African American: 11.1%.  Asian: 16.7%.  White - Caucasian: 72.2%. |
| Holmes, J. L., & Rockinson-Szapkiw, A. (2020). United States. | 282 | Range: 20-80 | Female: 72.3%  Male: 27.7% | Caucasians: 73.8%. |
| Jaksztat, S., Neugebauer, M., & Brandt, G. (2021). Germany. | 1,216 | Does not report | Female: 48.5  Males: 51.5% | Does not report |
| Jones, B. E., Combs, J. P., & Skidmore, S. T. (2019). United States. | 102 | Average age: 42 | Female: 71%  Male: 29% | White: 61%.  Black: 23%  Hispanic: 14%.  Other: 2% |
| Katz, R. (2018). Israel, Italy, Portugal and Switzerland. | 1,132 | Does not report | Does not report | Does not report |
| Lee, H., Chang, H., & Bryan, L. (2020). United States. | 210 | 20-29 years: 7%.  30-39 years: 33%.  40-49 years: 31%.  50-59 years: 25%.  60+ years: 4%. | Female: 77%  Male: 23% | Does not report |
| Litalien, D., & Guay, F. (2015). Canada. | 422 | Average age: 35.6 | Female: 45.5%  Male: 54.5% | Does not report |
| Lonka, K., Ketonen, E., Vekkaila, J., Cerrato Lara, M., & Pyhältö, K. (2019). Finland. | 664 | Average age: 39 | Female: 74.1%  Male: 25.9% | Does not report |
| Miller, A. N., & Orsillo, S. M. (2020). United States. | 436 | Average age: 30.51 | Female: 73.8%.  Male: 26.2% | Black racial identities: 42.7%  Hispanic/Latino racial identities: 26.6%  Multiracial identities: 24.7%. |
| Okahana, H., Klein, C., Allum, J., & Sowell, R. (2018). United States. | 2,667 | Average age: 27 | Female: 49.37  Male: 50.63 | Black/African American students: 49.33%.  Hispanic/Latino students: 46.21%  Other URM students: 4.47%. |
| Pyhältö, K., Peltonen, J., Castelló, M., & McAlpine, L. (2020). Finland, United Kingdom, Spain. | 2,426 | Average age: 36.54 | Female: 61.4%  Male: 38.6% | Does not report |
| Ribau, I., & Alves, M. G. (2018). United States. | 352 | Does not report | Does not report | Does not report |
| Rockinson-Szapkiw, A. J. (2019). United States. | 391 | Does not report | Female: 72  Male: 28% | Caucasians: 70.1%  African Americans: 13.8%  Asians: 4.6%  Hispanics: 8.2%  American Indian: 0.3%  Others: 3% |
| Rockinson-Szapkiw, A. J., Holmes, J., & Stephens, J. S. (2019). United States. | 232 | 30-39 years: 27.2%.  40-49 years: 6.6%. | Female: 75%  Male: 25%. | Caucasians: 77.2%.  African-Americans: 14.2%.  Hispanics: 5.2%.  Asians: 1.3%.  Asians: 0.4%.  American Indian: 1.7%. |
| Rockinson-Szapkiw, A. J., Spaulding, L. S., & Spaulding, M. T. (2016). United States. | 148 | 30-39 years: 35.1%.  40-49 years: 35.8%. | Female: 68.2  Male: 31.8%. | African Americans: 16.2%  Caucasians: 78.4%  Latinos: 2%  Asians: 2.7%  American Indian: 0.7% |
| Schwoerer, K., Antony, M., & Willis, K. (2021). United States. | 254 | Does not report | Female: 55.11%  Male: 44.89% | African-Americans: 30.3%. |
| Sverdlik, A., & Hall, N. C. (2020). Canada. | 3,004 | Average age: 30.39 | Female: 79.4%  Male: 20.6% | Caucasians:76.1%. |
| Sverdlik, A., Hall, N. C., & McAlpine, L. (2020). Canada. | 2,477 | Average age: 30.55 | Female: 76.7%  Male: 23.3% | United States: 49.5  Europe: 15.1%.  Canada: 9.7%.  United Kingdom: 9.1%.  Australia: 3.4%.  Other (e.g., Asia, Middle East): 2.9%. |
| Tao, K. W., & Gloria, A. M. (2019). United States. | 224 | Average age: 28.1 | Female: 100% | Non-Hispanic whites: 79.7%.  Asian / Asian American: 8.6%.  Hispanic/non-white: 5.9%.  Biracial / multiracial: 3.2%.  African American/Black: 0.9%.  American Indian/Alaska Native: 0.5%.  Two participants did not indicate their race or ethnicity: 0.9%. |
| Van der Linden, N., Devos, C., Boudrenghien, G., Frenay, M., Azzi, A., Klein, O., & Galand, B. (2018). Belgium. | 1,458 | Age at start of doctorate  25 or younger: 59%.  26 or older: 41%. | Female: 57%  Male: 43% | Does not report |
| Van Rooij, E., Fokkens-Bruinsma, M., & Jansen, E. (2021). Netherlands. | 839 | Average age: 28.9 | Female: 53%  Male: 47% | Does not report |
| Volkert, D., Candela, L., & Bernacki, M. (2018). United States. | 835 | 26-35: 32.6%.  36-45: 25.6%.  46-55: 22.2%. | Female: 91.3%  Male: 8.7% | Caucasians: 83.8%. |
| Wollast, R., Boudrenghien, G., Van Der Linden, N., Galand, B., Roland, N., Devos, C., De Clercq, M., Klein, O., Azzi, A., & Frenay, M. (2018). Belgium. | 1,509 | Does not report | Does not report | Does not report |

**Supplementary Material**

**Table 2**

*Scoping review results Part 1*

| **Author(s), year of publication and country** | **Objectives of the study** | **Population and sample** | **Study design and methodology** | **Type of sampling** |
| --- | --- | --- | --- | --- |
| Alkathiri, M. S. (2019). United States. | To assess the extent to which current Teaching and Learning doctoral students have developed self-authored perspectives and to examine whether the level of self-authorship varies with the number of years in the doctoral program. | 45 doctoral students enrolled in the Teaching and Learning program at a Midwestern university in the United States. | Observational, descriptive | Non-probabilistic convenience sampling |
| Ames, C., Berman, R., & Casteel, A. (2018). | To provide a preliminary assessment of students’ retention factors of engagement, communication, and isolation within an online doctoral program. | 698 doctoral students enrolled in EdD, PhD and DBA programs. | Observational, descriptive | Non-probabilistic convenience sampling |
| Bekova, S., & Dzhafarova, Z. (2019). Russia. | To evaluate the experiences of doctoral students balancing work and study and the main challenges they face.  To explore how factors such as students’ area of work, type of contract, and nature of work performed affect how they conceive of the educational process along with their specific learning outcomes and career prospects. | 2,020 doctoral students from 14 Russian universities (twelve universities participate in the Russian Academic Excellence Project "5-100" and two more are federal universities). | Observational, descriptive | Non-probabilistic convenience sampling |
| Boykin, T. F., & Walker, L. J. (2018). United States. | To determine whether faculty-student engagement was predictive of white doctoral students' overall experiences and whether it influenced students' belief that they would persist to completion. | 238 doctoral students attending HBCUs (Historically Black Colleges and Universities). | Correlational, non-experimental | Non-probabilistic convenience sampling |
| Byrom, N. C., Dinu, L., Kirkman, A., & Hughes, G. (2022). United Kingdom. | To assess the experience of doctoral students and identify factors that influence mental well-being and perceived stress. | 431 doctoral students in the United Kingdom. | Observational, descriptive | Non-probabilistic convenience sampling |
| De Clercq, M., Frenay, M., Azzi, A., Klein, O., & Galand, B. (2021). Belgium. | To identify the motivational profiles of doctoral students. | 461 PhD students enrolled at two Belgian universities. | Observational, analytical | Random sampling |
| Estrada, M., Zhi, Q., Nwankwo, E., & Gershon, R. (2019). United States. | To determine the effectiveness of professional development on motivation to persist in a scientific research career. | 101 biomedical doctoral students. | Observational, analytical | Random sampling |
| Gasa, V., & Gumbo, M. (2021). Ethiopia. | To analyze students’ perceptions of the dimensions of face-to-face support. | 120 Ethiopian doctoral students from a wide range of backgrounds. | Observational, descriptive | Non-probabilistic convenience sampling |
| Gruzdev, I., Terentev, E., & Dzhafarova, Z. (2020). Russia. | To provide new evidence on the supervision of doctoral students in Russian universities. | 2,034 doctoral students from 12 universities in Russia. | Observational, analytical | Non-probabilistic convenience sampling |
| Hands, A. S. (2020). United States, Canada. | To find out about the motivational influences of students to obtain a doctoral degree by analyzing the Academic Motivation Scale. | 18 doctoral students from programs in the United States and Canada. | Observational, descriptive | Non-probabilistic convenience sampling |
| Holmes, J. L., & Rockinson-Szapkiw, A. (2020). United States. | To define the integration of students in a distance learning doctoral program.  To develop and analyze the structure, validity and reliability of the Distance Doctoral Program Integration Scale. | 282 doctoral students enrolled in educational doctorate programs, in areas such as Curriculum and Instruction, Educational Leadership, Higher or Adult Education, and Instructional Design and Technology. | Observational, descriptive | Non-probabilistic sampling, snowball sampling |
| Jaksztat, S., Neugebauer, M., & Brandt, G. (2021). Germany. | To empirically evaluate the association of different pre-entry factors, institutional factors, and external factors with intention to leave doctoral studies. | 1,216 doctoral students in Germany. | Observational, analytical | Non-probabilistic convenience sampling |
| Jones, B. E., Combs, J. P., & Skidmore, S. T. (2019). United States. | To explore the relationships between pre-admission criteria and doctoral student performance ratings.  To develop a model to predict students’ persistence in an educational leadership doctoral program. | 102 doctoral students who entered the program in spring 2010 through summer 2013. | Observational, descriptive | Random sampling |
| Katz, R. (2018). Israel, Italy, Portugal and Switzerland. | To present quantitative results of a research study that evaluated crises reported by doctoral students while working on their degree. | 1,132 PhD students enrolled in five universities in Israel and three universities of technology in Western Europe. | Observational, analytical | Non-probabilistic convenience sampling |
| Lee, H., Chang, H., & Bryan, L. (2020). United States. | To explore how technological factors (TF) and relational factors (RF) predict student learning success (SLS). | 210 students from 26 online-based leadership doctoral programs in the United States. | Observational, descriptive | Non-probabilistic convenience sampling |
| Litalien, D., & Guay, F. (2015). Canada. | To provide a better understanding of persistence and completion of doctoral studies through the development and validation of a predictive model of dropout intentions. | 422 doctoral students enrolled in 66 different doctoral programs. | Observational, analytical | Non-probabilistic convenience sampling |
| Lonka, K., Ketonen, E., Vekkaila, J., Cerrato Lara, M., & Pyhältö, K. (2019). Finland. | To identify the personal profiles of doctoral students, which reflect their conceptions of themselves as writers. | 664 doctoral students at three faculties of the University of Helsinki: arts, medicine and educational sciences. | Observational, descriptive | Non-probabilistic convenience sampling |
| Miller, A. N., & Orsillo, S. M. (2020). United States. | To explore the relationship between racial and ethnic stressors, belongingness, acceptance, and valued living on the psychological functioning of graduate students from underrepresented minorities (URM). | 436 doctoral students who identified themselves as members of racial and ethnic groups underrepresented in doctoral programs in the United States. | Observational, descriptive | Non-probabilistic convenience sampling |
| Okahana, H., Klein, C., Allum, J., & Sowell, R. (2018). United States. | To analyze the likelihood of (URM) students completing the science, technology, engineering, and mathematics (STEM) PhD programs, their attrition rates, and the time spent until degree completion as a function of student demographic attributes, disciplines of study, and institutional participation. | 2,667 doctoral student records reported by 21 U.S. universities. | Observational, analytical | Non-probabilistic convenience sampling |
| Pyhältö, K., Peltonen, J., Castelló, M., & McAlpine, L. (2020). Finland, United Kingdom, Spain. | To explore cross-national variations in doctoral students' experiences by comparing the research interests of Finnish, British and Spanish doctoral students. | 2,426 PhD students in Finland, UK and Spain. | Observational, analytical | Non-probabilistic convenience sampling, snowball sampling |
| Ribau, I., & Alves, M. G. (2018). United States. | To study the characteristics of the doctoral population, in terms of its profile and main difficulties they might face to promote lower attrition rates and shorter completion times. | 352 PhD students enrolled at NOVA Lisbon University. | Observational, descriptive | Non-probabilistic convenience sampling |
| Rockinson-Szapkiw, A. J. (2019). United States. | To define the construct of academic-family integration in doctoral students, followed by the creation and validation of an instrument. | 391 doctoral students enrolled in Education, Counseling, Psychology and Business programs. | Observational, descriptive | Non-probability, snowball sampling |
| Rockinson-Szapkiw, A. J., Holmes, J., & Stephens, J. S. (2019). United States. | To examine whether personal factors (gender, race, age, marital status, and presence of children in the household) and program factors (stage in the doctoral journey, synchronous interactions, cohorts, and orientations) could predict doctoral program integration. | 232 students enrolled in online Doctorate in Education (EdD) programs. | Observational, analytical | Non-probability, snowball sampling |
| Rockinson-Szapkiw, A. J., Spaulding, L. S., & Spaulding, M. T. (2016). United States. | To examine the predictive relationship between institutional and integration variables on persistence. Integration variables included (a) academic integration, (b) social integration, (c) economic integration, and (d) family integration. Institutional variables addressed (a) financial support, (b) support services, and (c) program, curriculum and instruction. | 148 doctoral students enrolled in an online Doctor of Education (EdD) program. | Observational, analytical, descriptive | Non-probabilistic convenience sampling |
| Schwoerer, K., Antony, M., & Willis, K. (2021). United States. | To determine how stress and sources of social and institutional support affect students' perceptions of stress levels and balance in their lives.  To assess how access to social and institutional sources of support within doctoral programs affects the way students experience stress and balance in their lives while pursuing their doctoral program. | 254 doctoral students currently enrolled in traditional public administration programs, as well as in public affairs, public policy and public and non-profit management. | Observational, descriptive | Non-probabilistic convenience sampling |
| Sverdlik, A., & Hall, N. C. (2020). Canada. | To contribute to the literature on doctoral education by exploring the interaction between doctoral students' external programmatic factors (i.e., the study phase) and internal psychological processes (i.e., well-being and motivation) through large-scale quantitative analyses.  To examine whether the phase of the doctoral program (i.e., coursework, comprehensive exam, or dissertation phase) had an effect on doctoral students' levels of well-being (stress, depression, program satisfaction, and illness symptoms) and motivation (self-determined motivation and self-efficacy).  To assess the extent to which psychological processes in addition to academic identity, namely motivation and well-being, may also vary as a function of students' stage in their doctoral program. | 3,004 doctoral level students from 54 countries. | Observational, descriptive | Non-probabilistic convenience sampling |
| Sverdlik, A., Hall, N. C., & McAlpine, L. (2020). Canada. | To examine how the doctoral students’ integration into their scholarly community and their perceptions of self-worth shape their doctoral experiences. | 4,260 doctoral level students from all disciplines and all stages of the doctoral process. | Observational, analytical, descriptive | Non-probabilistic convenience sampling |
| Tao, K. W., & Gloria, A. M. (2019). United States. | To examine the association between impostorism and doctoral students' perceptions of their self-efficacy, their research and training environment, and their attitudes toward academic persistence. | 224 doctoral students from a Midwestern university. | Observational, descriptive | Non-probabilistic convenience sampling |
| Van der Linden, N., Devos, C., Boudrenghien, G., Frenay, M., Azzi, A., Klein, O., & Galand, B. (2018). Belgium. | To develop and validate Doctorate-related Need Support and Satisfaction short scales (D-N2S). | 1,458 PhD students from two Belgian universities. | Observational, descriptive | Non-probabilistic convenience sampling |
| Van Rooij, E., Fokkens-Bruinsma, M., & Jansen, E. (2021). The Netherlands. | To investigate which supervisory, psychosocial and project characteristics are related to doctoral students’ success, where doctoral success is measured as being satisfied with the overall doctoral trajectory, being on schedule (perception of progress), and considering leaving the program (intent to drop out). | 839 PhD students at a university in the Netherlands. | Observational, descriptive | Non-probabilistic convenience sampling |
| Volkert, D., Candela, L., & Bernacki, M. (2018). United States. | To examine how the effects of environmental stressors predict students' intention to leave their current doctoral program of study. | 835 doctoral students in nursing at one university in the United States. | Observational, descriptive | Non-probabilistic convenience sampling |
| Wollast, R., Boudrenghien, G., Van Der Linden, N., Galand, B., Roland, N., Devos, C., De Clercq, M., Klein, O., Azzi, A., & Frenay, M. (2018). Belgium. | To investigate the factors associated with doctoral completion in students outside the USA through the use of univariate and multivariate analyses. | 1,509 PhD students who started their PhD process during the academic year 2005-2006 or 2006-2007 from two universities in Belgium. | Observational, descriptive | Non-probabilistic convenience sampling |

**Supplementary Material**

**Table 3**

*Scoping review results Part 2.*

| **Author(s), year of publication and country** | **Main results** | **Instruments used** | **Relevant findings** |
| --- | --- | --- | --- |
| Alkathiri, M. S. (2019). United States. | 1. The results showed high reliability, as well as significant correlations between all constructs.  2. Measures on all three constructs for participants who had been in the PhD program for one to two years were lower than the means for participants who had been in the program for three to six years. | Doctoral Students’ Self-Authorship Questionnaire (DSSAQ). | The sample of students who had been longer in the doctoral program showed greater agreement with self-authorship perspectives on the three dimensions Epistemological, Intrapersonal, and Interpersonal. The comparisons were not statistically significant on all three dimensions; therefore, it cannot be concluded that time in the PhD has an effect on individuals' authorship development. |
| Ames, C., Berman, R., & Casteel, A. (2018). | 1. Preliminary results indicate that private doctoral workspaces did not change the perception of isolation among participants 2.  2. There is a perception that the private PhD workspace improved effective communication with dissertation committees by PhD students over time | A survey was used to identify doctoral student perceptions of private doctoral workspaces' capacity to reduce isolation and increase communication between doctoral students and their dissertation committees, and to measure how frequently doctoral students engaged with the broader doctoral community. | 1. The implementation of online workspaces for doctoral students addressed factors experienced in online doctoral programs. 2. The introduction of private doctoral workspaces significantly improved doctoral students’ perceptions of more effective communication with their dissertation committees. 3. Perceptions of isolation remained unchanged with the introduction of the technology. |
| Bekova, S., & Dzhafarova, Z. (2019). Russia. | 1. Working on a full-time contract outside the university while studying for a PhD is a factor that can materially harm their prospects for success.  2. Those students who choose to work while enrolled in a doctoral education program are likely to exhibit much clearer directions for their future careers  3. Cohorts of aspiring PhD candidates who are employed inside and outside the university tend to report sharply polarized career plans. | Survey of 2,020 PhD students at 14 leading Russian universities on their motives to enter a doctoral program, how they plan to complete their studies and find employment. | Balancing work and study can benefit both the academic performance and professional experiences of doctoral students, but only when their dissertation research topic is closely aligned with what they do in the workplace. |
| Boykin, T. F., & Walker, L. J. (2018). United States. | 1. White doctoral students attending HBCUs who were highly internally involved with faculty and interacted a great deal with faculty advisors showed high levels of interpersonal interaction with faculty.  2. The results of this study indicated that white doctoral students had overall positive experiences while attending an HBCU and were convinced that they would meet all the requirements to obtain the doctoral degree.  3. Faculty engagement, characterized as faculty-student engagement, is indeed a critical factor for the experiences and perceived persistence of White doctoral students attending HBCUs. | The researcher developed a survey by extracting selected items from the Survey of Doctoral Student Finances, Experiences and Achievement (Nettles & Millet, 2006), the Survey on Doctoral Education and Career Preparation (Golde & Dore, 2001), and the Survey of Earned Doctorates (National Opinion Research Center, 2006). | The results indicated external engagement, i.e., social components to student success outside of the academic program and student research practices was the best predictor of optimal experiences and increased self-confidence to complete the program. |
| Byrom, N. C., Dinu, L., Kirkman, A., & Hughes, G. (2022). United Kingdom. | 1. Respondents gave positive reports about their supervisory relationship and identified feeling confidently prepared for their job.  2. Family support, good general health, sleep, and low levels of self-deprecation predicted stronger mental well-being and lower levels of stress  3. Students who were confident about their future career and felt well prepared for their studies were less stressed and those who were achievement-oriented had better mental well being | 1. The survey included items relating to demographics (age, gender, ethnicity, sexuality) and student status (university of study, format and subject of study and student status, i.e., home, EU or international student).  The 7-item Short Warwick Edinburgh Mental Wellbeing Scale (SWEMWBS; Stewart-Brown et al., 2009).  3. Impostor syndrome was assessed using the five-item self-depreciation subscale on the Perceived Fraudulence Scale (PFS; Kolligian & Sternberg, 1991).  4. The 12-item Multidimensional Scale of Perceived Social Support (MSPSS; Zimet et al., 1988) was used to assess social support.  5. To assess motivation, enthusiasm and preparation for academic work, we used the 10-item Achievement Orientation Scale, from the Berkeley Report (Panger et al., 2014). | Stress and mental well-being among doctoral researchers are influenced by factors including health, sleep, and social support. In general, students feel well supported by their supervisors, but respondents do not identify the same support from the department and faculty.  Career confidence among doctoral researchers is low and addressing this issue might be important for their mental well-being. In this respect, orientating students towards achievement and minimizing their feelings of self-deprecation are vitally important.  Efforts to increase the confidence of PhD researchers and provide more rigorous academic scaffolding to reduce fear of failure and develop self-efficacy can have a positive impact on mental well-being. |
| De Clercq, M., Frenay, M., Azzi, A., Klein, O., & Galand, B. (2021). Belgium. | 1. Affinity and competence are related to the actual completion of doctoral studies two years later.  2. Intention to persist is strongly related to burnout, sense of progress, and ownership of the thesis.  3. The strongest correlate of PhD completion is the intention to persist. | Motivational Self-Perceptions were assessed through 12 items retrieved from the Doctorate-related Need Support and Satisfaction (DN-2S) short scales (Van der Linden et al., 2018).  Supervisor Support was evaluated with the validated scale of D-N2S (Van der Linden et al., 2018)  3. Exhaustion was assessed through 10 items, inspired by the measure adapted from Hunter and  Devine (2016)  4. Appropriation of the Project referred to the idea of ownership of the thesis project by the doctoral student and was assessed through a scale inspired by Devos et al.'s findings (2017).  5. Intention to Persist assessed the strength of participants' intention to persist in their PhD and complete it with items based on the studies by Galand and Hospel (2015) and by Litalien and Guay (2015). | 1. Particular motivational profiles were identified among doctoral students.  2. An important variation in the doctoral process was revealed among the profiles.  3. The direct effect of the intention to persist in the completion of the doctorate was confirmed.  4. Partial evidence that supports the idea that a supervisor does not have the same effect on all doctoral students. |
| Estrada, M., Zhi, Q., Nwankwo, E., & Gershon, R. (2019). United States. | No significant direct relationships were found for HU or majority students between measures of social support at baseline and intentions to persist in science one year later 2.  2. The results showed significant relationships between scientific identity and friends and family support and professional network support for the most represented students  3. The types of social support related to scientific identity were largely different for HU doctoral students and the majority  4. Scientific identity significantly mediated the effect on the ratio of instrumental support predicting persistence for HU students. Mediation was insignificant for majority students.  5. Scientific identity has a significant mediated effect on friend-family support relationship that predicts persistence | The Instrumental, Psychological and Professional Network Support were each assessed through Likert scales based on the NIH mentoring guideline (referencia actualizada).  Friend and Family support was measured with a scale adapted from Lent et al’s work (2013).  Science Identity and Persistence were both assessed through measures adapted from Estrada et al’s investigation (2011). | Results indicated that scientific identity significantly mediated the relationship between professional network support and persistence one year later for most students. For HU students, scientific identity mediated the relationship between instrumental, psychosocial, friends and family support and persistence one year later. |
| Gasa, V., & Gumbo, M. (2021). Ethiopia. | 1. Age proved to be a statistically significant effect on perceptions of all dimensions of student support (with older student perceptions being the least positive) 2.  2. The number of visits to the Ethiopian campus proved to have a significant impact on perceptions regarding the accommodation of student needs  The biographical effects of age, registration period, and visits to the Ethiopian campus were reliably verified to statistically significantly affect perceptions regarding the value of student support offered primarily to Ethiopian doctoral students. | A closed-ended questionnaire with 35 questions, of which 26 offered 5-point agreement Likert rating. | Experiences of the five dimensions investigated in the study were deemed as positive by the student-respondents |
| Gruzdev, I., Terentev, E., & Dzhafarova, Z. (2020). Russia. | 1. Prevalence of supervisor categories differs significantly by year of study.  2. The prevalence of categories also differs significantly according to the field of study.  3. There are statistically significant differences in the reported willingness to change supervisors among the different supervisory styles | The survey section on supervision included questions concerning (1) the functions performed by the supervisor, (2) aspects of communication with the supervisor (frequency, difficulties, communication beyond the work on dissertation), and (3) satisfaction with the supervisor (desire to change the supervisor). | For the most problematic category, characterized by not providing assistance to doctoral students and labeled "non-intervention supervisors", doctoral students reported the lowest level of satisfaction and the highest expected time to degree.  The large proportion of doctoral students who are satisfied with non-intervention supervisors may evidence the presence of a disengagement pact between doctoral students and supervisors in Russian universities.  Supervisors with the highest level of satisfaction from doctoral students and the shortest expected time to degree were termed "superheroes" and "mentors" |
| Hands, A. S. (2020). United States, Canada. | Participants were motivated to pursue the PhD because of:  - the perceived enjoyment and satisfaction of learning and exploring new information reflected in the correspondence defined with the scale items related to intrinsic motivation to know  - obtaining the degree presented an opportunity to excel academically and achieve a challenging personal goal demonstrated by a definite correspondence with items of intrinsic achievement motivation  - perceived value in the Ph.D., viewing doctoral study as necessary career preparation, as noted by moderate correspondence with items reflecting identified regulation; and  - D. study was an opportunity to immerse oneself in the work of key thinkers in their chosen field and to experience the positive feelings associated with such academic engagement, as evidenced by the correspondence  moderate with the items of the intrinsic motivation subscale for stimulation | 1. The General Causality Orientation Scale.  The Aspirations Index assesses an individual's aspirations in 7 categories.  3. Early motivation instrument based on self-determination theory (SDT).  4. Academic Motivation Scale (AMS-C 28). | Doctoral students are motivated by several types of intrinsic motivation, as well as identified regulation, a type of extrinsic but autonomous motivation. |
| Holmes, J. L., & Rockinson-Szapkiw, A. (2020). United States. | Three dimensions were identified:  1. Faculty integration is the level of satisfaction with the nature and quality of interactions between academic and non-academic students and faculty that take place during the distance PhD program  2. Student integration is the level of satisfaction with the nature and quality of student-student academic and non-academic interactions that take place during the distance doctoral program.  3. Curriculum integration is the level of satisfaction with the quality and relevance of the curriculum in the distance doctoral program. | Distance Doctoral Program Integration Scale (DDPIS). | The results indicated a three-factor structure (i.e., faculty integration, student integration, and curricular integration).  The 32-item instrument is valid and reliable in measuring the programmatic integration of doctoral students studying at a distance. |
| Jaksztat, S., Neugebauer, M., & Brandt, G. (2021). Germany. | 1. Female doctoral candidates drop out at higher rates than their male counterparts 2.  Individuals whose parents have earned a doctorate were found to have significantly lower dropout rates compared to individuals from non-academic families.  It is evident that better school grades and university grades are negatively associated with dropout.  4. Compared to the humanities benchmark group, people in mathematics/natural sciences, medicine, and engineering have lower attrition rates  5. Receiving a scholarship is associated with a lower risk of attrition even when controlling for performance indicators  6. Close contact with the supervisor and exchange with other doctors are associated with lower attrition rates.  7. We find strong support for the assumption that people who are well-integrated into academia are less likely to drop out  8. The analysis confirms that doctoral students who already have children or become parents during their doctoral studies drop out at a higher rate.  9. Receiving a permanent job during the PhD increases the probability of attrition, but in the fully specified model, the effect of a permanent job is no longer significant | The studies gathered longitudinal data using event history modelling, observing doctoral students in multiple disciplines and a wide range of universities.  The panel survey consists of three waves that were conducted approximately 1 year, 5 years, and 10 years after graduation. | 1. Women are more likely to dropout than men. 2. The probability of dropping out strongly depends on the discipline and the availability of a scholarship. 3. A close contact with the supervisor and exchange with other PhDs are associated with a lower dropout probability. 4. Having children increases dropout rates. |
| Jones, B. E., Combs, J. P., & Skidmore, S. T. (2019). United States. | 1. Value was found in the use of the GPA and GRE in admission decisions.  2. A single model may not be effective for all admissions. | The overall student performance was gauged by a rating form provided to doctoral faculty members who had taught a majority of the students in at least one course. Faculty members were asked to rate the overall performance of the students they had taught with a holistic score ranging from 1 to 5. | GRE-Q and undergraduate GPA are useful predictors of doctoral student persistence.  GRE-V and graduate GPA are also useful predictors, but differently for students of color and white students.  Separate models were created because they realized the inability of a single model to adequately predict the propensity of students of color and white students to remain in the program. |
| Katz, R. (2018). Israel, Italy, Portugal and Switzerland. | 1. Statistical analysis showed no significant difference between the percentage of internal and external students reporting crises among Israeli SS&H students. E.  2. Failed to test the hypothesis that part-time external students report more crises than full-time internal students.  3. Students who reported a balanced investment of 30 to 60 hours per week in their doctoral research reported the lowest percentage of crisis  4. Very different patterns of meetings between students and advisors were found in the "soft sciences" as opposed to the "hard sciences."  5. Students who responded that they enjoy their life as a doctoral candidate reported significantly less crisis than candidates who responded that they did not enjoy their life as a candidate and were disappointed in the program. | The survey included 26 questions of which seven questions are included in the current analysis: three that relate to crises and four that relate to our research questions. | 1. Separate models for students of color and White students most accurately predicted program performance, indicating that a one-size fits all approach was not optimal. 2. The GRE-Q and undergraduate GPA were useful predictors of doctoral student persistence. 3. The GRE-V and graduate GPA were also useful predictors but differentially so for students of color and White students. |
| Lee, H., Chang, H., & Bryan, L. (2020). United States. | 1. All sub-factors of technological factors (TF) and relational factors (RF) significantly predicted student learning success (SLS).  2. The technology sub-factors - usability, flexibility, and ease of use - and a separate student-non-faculty relational sub-factor did not significantly predict student learning success (SLS).  3. The relational sub-factors: student-student relationship and student-teacher relationship, were statistically significant with respect to the effects on student learning success significantly (SLS). | The 73-item Online Learning Success Scale (OLSS) was constructed and administered online to collect self-reported data on three primary variables: student learning success (SLS), relational factors (RF), and technological factors (TF). | 1. Relational Factors and Technological Factors both separately and together predict Student Learning Success. 2. While all dimensions of Relational Factors and Technological Factors are significant predictors of Student Learning Success, the student-faculty relationship was the strongest one. |
| Litalien, D., & Guay, F. (2015). Canada. | 1. Students who perceived themselves as more competent were more likely to complete their doctoral program 2.  2. The quality of the relationship with the advisor and faculty is relevant.  3. Differences were found by gender, citizenship status and type of program, mainly in favor of natural science students.  4. Differences in citizenship status were mainly related to financial aspects  5. Non-citizens also showed more controlled regulation.  6. The more progress they make in their PhD program and the more often they present at research conferences and related events, the less likely students are to consider dropping out of their program  7. Both types of regulation predicted perceived competition. | 1. Doctoral students’ motivation was assessed with the Motivation for PhD Studies scale (Litalien et al., 2015.  2. Perceived competence was measured with the competence subscale of the Balanced Measure of Psychological Needs scale (BMPN; Sheldon & Hilpert, 2012).  3) Support for psychological needs was assessed through three different scales (Rochester Assessment Package for Schools, Connell & Wellborn, 1991; Markland & Tobin, 2010; Learning Climate Questionnaire, Williams & Deci, 1996). | 1. The model showed a five-factor first-order structure and a two-factor higher-order structure, scale reliability, and convergent and discriminant validity. 2. Complete measurement invariance was supported across gender, citizenship status, program type, age, and program progression. |
| Lonka, K., Ketonen, E., Vekkaila, J., Cerrato Lara, M., & Pyhältö, K. (2019). Finland. | 1. Knowledge transformation was clearly the variable that most differentiated the profiles, and its effect size on the cluster solution was very strong  2. In terms of blocks, perfectionism, innate ability, and productivity, the differences between the profiling variables were not as decisive, although we did find some statistically significant pairwise differences  3. Procrastination did not differentiate profiles.  4. The results revealed that lack of interest varied significantly as a function of writing profile | 1. The Writing Process Questionnaire (Lonka et al. 2014; see https://doi.org/10.17239/jowr2014.05.03.1) consisted of six sub-scales for measuring blocks, knowledge transformation.  2. They measured experienced well-being using the MED NORD questionnaire, modified for the context of doctoral studies (Lonka et al. 2008; Stubb et al. 2011, 2012).  They measured the doctoral students' perceptions of the learning environment (Dahlin et al. 2005) using 13 Likert scale items, including feedback. | Although problems in writing are quite common, epistemic beliefs may be even more decisive in terms of successful research writing.   1. Three writing profiles were identified: *Growth-Transforming*, *Ambivalent* , and *Fixed-Blocking* . 2. The *Fixed-Blocking group* reported most frequently a lack of interest and receiving the least feedback. 3. The *Growth-Transforming group* was the most with their studies, whereas *Fixed-Blocking group* was the least satisfied. 4. Three writing profiles were identified: *Growth-Transforming*, *Ambivalent* , and *Fixed-Blocking* . 5. The *Fixed-Blocking group* reported most frequently a lack of interest and receiving the least feedback. 6. The *Growth-Transforming group* was the most with their studies, whereas *Fixed-*Blocki*ng group* was the least satisfied. |
| Miller, A. N., & Orsillo, S. M. (2020). United States. | 1. URM doctoral students who had been exposed to higher rates of racial stressors and microaggressions in their graduate programs reported higher rates of depression, anxiety, and stress, although the magnitude was weak.  2. A lower sense of personal belonging in one's doctoral program was found to be associated with higher rates of depression, anxiety and stress.  Both participation in a valued life and a self-reported accepting stance toward challenging internal experiences uniquely contributed to all three aspects of psychosocial functioning (depression, anxiety, and stress) over and above the effects of exposure to racial stressors, microaggressions, and membership. | Participants completed the Schedule of Racist Events, Racial and Ethnic Microaggressions Scale, Campus Connectedness Scale, Valued Living Questionnaire, Philadelphia [Mindfulness](https://www.sciencedirect.com/topics/psychology/mindfulness) Scale, and the Depression, Anxiety, and Stress Scales. | 1. Racial and ethnic microaggressions and stressors were positively associated with [psychological distress](https://www.sciencedirect.com/topics/psychology/psychological-distress) 2. Belongingness was negatively associated with psychological distress. 3. However, both acceptance of internal experiences and values-based living predicted psychological functioning (depression, anxiety, and stress) over and above the negative effects of racial and ethnic stressors and low perceived belongingness. |
| Okahana, H., Klein, C., Allum, J., & Sowell, R. (2018). United States. | 1. For both females and males, Hispanic/Latino students were more likely than their Black/African American counterparts to have earned their doctorate in science, technology, engineering, and mathematics (STEM) within ten years.  2. Regarding the effects of previous Master's degrees, there were no statistically significant covariates among those with previous Master's degrees.  3. Compared to those who earned their degrees in the physical and mathematical sciences, engineering PhD recipients had a shorter time to degree, while social and behavioral science students took slightly longer to complete. | No instrument reported (Data bases). | 1. Hispanic/Latino students and students from other underrepresented groups complete at higher rates than their Black/African American counterparts. 2. Prior master's degrees and institutional participation in doctoral completion programs are positively correlated with STEM doctoral completion. |
| Pyhältö, K., Peltonen, J., Castelló, M., & McAlpine, L. (2020). Finland, United Kingdom, Spain. | Some differences were detected between Spanish, Finnish and British PhD students research interest:  1. Spanish PhD students showed a higher research interest than UK students or Finnish students 2.  2. Spanish PhD students also showed considerably higher levels of instrumental interest compared to their counterparts in the UK and Finland.  3. UK students, on the other hand, clearly showed the lowest levels of interest in development compared to Spanish and Finnish PhD students.  4. An additional comparison showed that Spanish PhD students experienced less cynicism than UK students.  5. Finnish PhD students reported slightly less burnout than either UK or Spanish students.  6. Spanish students were more satisfied with their doctoral studies than Finnish students. | Participants responded to the Doctoral Experience survey (Pyhältö et al., 2016) | 1. Spanish students maintained higher levels of researcher and instrumental interest compared to UK and Finnish students. 2. Finnish students showed the lowest levels of instrumental interest while UK students combined the lowest level of developmental interest with the highest level of cynicism. 3. Interest was a determinant of experienced burnout, cynicism, study satisfaction, and reduced dropout risk in all three contexts. |
| Ribau, I., & Alves, M. G. (2018). United States. | 1. ISEGI/Nova IMS students used regular time.  2. ITQB and NMS/FCM students need one year, longer than the usual time. In areas, such as science and engineering, students take a shorter time to complete the doctorate (FCT, ITQB) than social science students (FCSH).  3. There are more female than male PhD completers, only in economics at Nova SBE and ISEGI/NOVA IMS it is found that the number of female PhD completers was lower than male. | The data were collected from a public national annual survey applied to all higher education institutions in Portugal and from an institutional document on the programs offered by NOVA university. | 1. Women were more likely to complete their studies, however, their completion time was longer. 2. The relationship established between the student and the supervisor is related to gender and offers psychological advantages for male students and disadvantages for female students. 3. Internationalization is slowly increasing due to the number of studies published and the increase in the number of professors and students. 4. The number of scholarship holders is highlighted, as well as the fact that most of the students are not workers and therefore need economic support from other sources. |
| Rockinson-Szapkiw, A. J. (2019). United States. | The Principal Components Analysis (PCA) demonstrated that the Doctoral Academic-Family Integration Inventory (DAFII) is valid and reliable and consists of three subscales. | Expert panel and PCA to examine the validity of the Doctoral Academic-Family Integration Inventory (DAFII) on a sample of doctoral students. | 1. This research provides a psychometrically sound instrument to advance research on academic-family integration. 2. The DAFII also provides a tool for expanding research on persistence. |
| Rockinson-Szapkiw, A. J., Holmes, J., & Stephens, J. S. (2019). United States. | 1. Personal factors played an important role in explaining program integration.  2. The regression model combining personal and program factors was significant and accounted for 26.8% of the variance in students’ program integration.  3. Several variables made significant individual contributions to explaining program integration, including gender, race, cohort participation, and participation in synchronous meetings. | Scale of Distance Doctoral Program Integration Scale (DDPIS; Holmes & Rockinson-Szapkiw, 2019).  Data were gathered through both an online survey consisting of validated instruments and researcher developed questions.  Program integration, the criterion variable, was measured with the Distance Doctoral Program Integration Scale (DDPIS; Holmes & Rockinson-Szapkiw, 2019). | 1. Both personal (i.e., gender, race, age, marital status, and presence of children in the home) and program variables (i.e., stage in the program, the presence of synchronous interaction, use of a cohort model, and participation in an orientation) influence program integration of online doctoral students. 2. The variables of gender, race, being part of a cohort, and participating in synchronous meetings made significant individual contributions in explaining the variance in student satisfaction with program integration. |
| Rockinson-Szapkiw, A. J., Spaulding, L. S., & Spaulding, M. T. (2016). United States. | The results of the direct logistic regression analysis showed that the entire model, including all institutional and integration variables, significantly predicted whether an online PhD candidate would persist in the dissertation phase of their program.  Support services; program, curriculum, and instruction; academic integration; faculty connectivity; and family integration made significant individual contributions to the model explaining persistence.  Family integration was a strong predictor of persistence.  Online doctoral candidates with good family integration were more than twice as likely to persist through the dissertation phase of the doctoral process compared to students with poor family integration.  Greater satisfaction with support services and programs, curriculum, and instruction also significantly increased the likelihood that a student would continue in dissertation courses in their online program. | Archival data from doctoral students enrolled in an online Doctor of Education program. | 1. The entire model, including all institutional and integration variables, significantly predicted whether or not online doctoral students would persist to the candidacy stage of the program. 2. Support services; program quality, curriculum and instruction; academic integration; social integration with faculty; and family integration each individually contributed to explaining the likelihood of online doctoral persistence. |
| Schwoerer, K., Antony, M., & Willis, K. (2021). United States. | 1. The direct effect of emotional support on work-life conflict is negative, but not statistically significant.  2. The effect of institutional sources of support and find that both academic program support and professional support are strongly and negatively associated with work-life conflict.  3. Stress is strongly and positively associated with perceptions of work-life conflict.  4. Doctoral students who perceive greater support from an academic mentor report less stress and fewer work-life conflicts.  5. Institutional sources of support and find that both academic program support and professional support also reduce the amount of stress students report experiencing (consistent with H3c and H3d)."  6. The relationship between emotional support and work-life conflict is completely mediated by perceptions of stress which consists of regressing emotional support on work-life conflict and, at the same time, managing stress.  7. When mediation is assessed, the effect of academic mentor support becomes statistically insignificant while stress remains significant, indicating complete mediation. | Scales were employed to measure work-family conflict (WFC) and family-work conflict (FWC).  The measures used for emotional support and academic mentor support were adapted from Pascarella and Terenzini’s (1980) scales for peer-group and faculty interaction.  Stress was measured using Cohen et al.’s (1983) Perceived Stress Scale (PSS). | Four sources of support (emotional support, academic mentor support, program support, and professional support) were found to be instrumental in decreasing students' stress and perceptions of conflict between their academic and non-academic lives. |
| Sverdlik, A., & Hall, N. C. (2020). Canada. | 1. Well-being: analyses revealed significant phase effects on perceived stress and satisfaction with the program.  2. Motivation: three of the five subscales of self-determined motivation were significantly different between the PhD phases, including integrated motivation, identified motivation, and introjected motivation.  3. A significant difference was observed between the coursework and comprehensive examination phases on introjected motivation, showing that introjected motivation is lower in the first doctoral phase and higher in the later phases. No significant difference was observed between the comprehensive examination and dissertation phases.  4. Although self-efficacy did not differ significantly between the coursework phase and comprehensive examination phase, significant differences were observed between the first two phases and the dissertation phase, with students in the dissertation phase reporting the highest levels of self-efficacy. | 1. A 17-item demographic questionnaire assessed information relevant to participants' identity and background information.  2. The short version of the Center for Epidemiological Studies Depression Scale (Andersen E. M., et al., 1994) was used to assess depressive symptoms.  3. Stress was evaluated with a 10-item Strain scale (Cohen & Williamson, 1988) assessing the frequency of strenuous thoughts and feelings.  4. Satisfaction with the program was evaluated using the 5-item Satisfaction with Life scale (Diener, Emmons, Larsen, & Griffin, 1985).  5. An 8-item Health and Illness Symptoms scale, adapted from Cohen and Hoberman (1983) was used to assess physical health.  6. The motivation for PhD studies scale (Litalien et al., 2015) was used to evaluate five types of motivation specific to doctoral education.  7. An 11-item self-efficacy scale developed for this study assessed participants perceived confidence in their ability to successfully complete multiple specific graduate school tasks. | 1. Doctoral students reported the highest well-being and internal motivation during the coursework phase, while the comprehensive examination phase proved to be the most challenging for most students, as indicated by the lowest well-being and motivation scores. |
| Sverdlik, A., Hall, N. C., & McAlpine, L. (2020). Canada. | 1. Doctoral students' perceptions of belonging to their academic community corresponded with lower levels of impostor syndrome.  2. Perceived belongingness is a negative predictor of imposter syndrome which, in turn, predicted higher levels of depression, stress, and symptoms of illness.  3. The imposter syndrome significantly measured the relationship between perceived academic belongingness and the three outcome variables assessing psychological well-being.  4.Perceived academic belonging negatively predicts imposter syndrome.  5.Imposter syndrome predicts increases in depression, stress, and disease symptoms in the sample of doctoral students. | 1. 17-item demographic questionnaire solicited information relevant to participants' demographic characteristics.  2. The perceived scholarly belongingness measure was adapted from Adams et al.'s Professional Identity scale to assess perceptions of membership within one's scholarly community.  3. Participants completed a 10-item imposter syndrome scale (Clance, 1985).  4. The short version of the Center for Epidemiological Studies Depression Scale (CESD10; Radloff, 1977) was used to assess depressive symptoms.  5. Perceived stress was evaluated with a 10-item Strain scale from Cohen and Williamson (1988).  6. Students' physical health was assessed with an 8-item Health and Illness scale, adapted from Cohen and Hoberman (1983). | 1) Perceived belongingness is a negative predictor of imposter syndrome which, in turn, predicted higher levels of depression, stress, and symptoms of illness.  2) The imposter syndrome significantly measured the relationship between perceived academic belongingness and the three outcome variables assessing psychological well-being.  3) Perceived academic belongingness negatively predicted impostor syndrome five months later, and impostor syndrome, in turn, predicted increases in depression, stress, and disease symptoms among doctoral students. |
| Tao, K. W., & Gloria, A. M. (2019). United States. | 1. A negative and significant association was found between impostorism and students' perceptions of self-efficacy, the research-training environment, and persistence attitudes.  2. Impostorism indirectly affected the persistence attitudes of female STEM doctoral candidates through its effect on academic self-efficacy and perceptions of the research-training environment.  3. When the number of women in a STEM doctoral program increased, the association between impostorism and academic persistence attitudes increased. | 1. Demographic questionnaire, which included questions on age, race-ethnicity, area of study, year in PhD program, and perceived percentage of women in the PhD program.  2. Clance Impostor Phenomenon Scale (CIPS; Clance & Imes, 1978).  3. Persistence/Voluntary Dropout Decision Scale (P/VDD; Pascarella & Terenzini, 1980).  4. The 16-item Likert-based Graduate Student Self-Efficacy scale (GSSE; Alvarez, 1992).  5. Research Training Environment Scale-Revised Short scale (RTES-R-S; Gelso, Mallinckrodt, & Judge, 1996; Kahn & Miller, 2000). | 1) Higher levels of impostorism would be linked to a more somber outlook toward completion of a STEM-related doctoral program, lower self-efficacy, and negative perceptions of the doctoral environment.  2) Correlations between impostorism, self-efficacy, research training environment and persistence ranged from medium to large. |
| Van der Linden, N., Devos, C., Boudrenghien, G., Frenay, M., Azzi, A., Klein, O., & Galand, B. (2018). Belgium. | 1. Competency satisfaction explains most of the variance in doctoral persistence intentions.  2. The average correlations between the dimensions of need satisfaction and doctoral persistence intentions were higher than those of need satisfaction.  3. The need for support and the satisfaction of needs are made up of three separate but related dimensions~~.~~ | Self-report scales of Doctorate-related Need Support and Need Satisfaction (D-N2S). | 1. The scales showed satisfactory psychometric properties of reliability, as well as construct, criterion-related, known-group, predictive and face validity. 2. The scales exhibited satisfactory [psychometric](https://www.sciencedirect.com/topics/psychology/psychometrics) properties of reliability as well as construct, criterion-related, known-groups, predictive, and face kinds of validity. All but one dimension (i.e., relatedness satisfaction) was positively related to doctoral persistence intentions and all but two dimensions (i.e., [autonomy](https://www.sciencedirect.com/topics/social-sciences/autonomy) support and relatedness satisfaction) were negatively related to actual dropout. As expected, doctoral students in social sciences and humanities reported higher levels of autonomy support and autonomy satisfaction but lower levels of relatedness satisfaction than doctoral students in sciences and technology or health sciences. 3. Doctoral students in the social sciences and humanities reported higher levels of autonomy support and satisfaction with autonomy, but lower levels of satisfaction with relatedness than PhD students in science and technology or health sciences. |
| Van Rooij, E., Fokkens-Bruinsma, M., & Jansen, E. (2021). Netherlands. | 1. Gender, nationality, and project phase mattered: men were more satisfied, European PhD candidates and PhD candidates in their final years less satisfied.  2. Gender was a significant predictor of risk of dropout: men less frequently considered abandoning their PhD project.  3. Doctoral candidates who indicated that they perceived less academic support and who reported that their supervisor had high expectations more often considered resigning.  4. Doctoral candidates who reported a lower quality relationship with their day-to-day supervisor more often considered resigning. | The supervisor support scales, i.e., autonomy support, personal support, academic support, and supervisor availability, were based on scales developed by Overall, Deane, and Peterson (2011).  The scales regarding psychosocial factors - formal relationships, informal relationships, and sense of belonging - were based on scales that Meeuwisse, Severiens, and Born (2010). | 1. Experienced workload was negatively related to satisfaction and progress and positively related to intentions to leave the program. 2. The quality of the supervisor-PhD candidate relationship, sense of permanence of the PhD candidate, the amount of freedom in the project, and working on a project closely related to the supervisor's research were positively related to satisfaction and negatively related to intentions to leave the program. |
| Volkert, D., Candela, L., & Bernacki, M. (2018). United States. | 1. Support problems, significantly predicted intention to abandon the program.  2. Program stressors significantly predicted intention to drop out of the program.  3. As stressors related to differing expectations among faculty / staff increased, program stressors significantly predicted intention to drop out of the program.  4. Advisor and student and the isolating / overwhelming nature of the program, also increased the intention to drop out of the program of study. | The 57-item Nursing Doctoral Stressors and Motivation questionnaire was developed from previous instruments used to assess students in science, technology, engineering, and mathematics programs (Perez et al., 2013). | 1. Stressors related to program problems, primarily student-faculty/advisor relationships, significantly predict intention to leave. As program stressors increase, so does intention to leave. |
| Wollast, R., Boudrenghien, G., Van Der Linden, N., Galand, B., Roland, N., Devos, C., De Clercq, M., Klein, O., Azzi, A., & Frenay, M. (2018). Belgium. | 1. The relationship between PhD success/dropout and gender was not statistically significant. However, success rates tended to be higher among men than among women.  2. The relationship between PhD success / dropout and nationality was significant. the Belgian nationals have a higher PhD completion rate compared to non-EU nationals  3. The relationship between PhD success/dropout and marital status was significant, suggesting that the success rate is higher among married persons  4. The relationship between PhD success/dropout and age at registration is significant: younger PhD students have a higher PhD completion rate  5. The relationship between PhD success/dropout and master's degree was significant: a higher master's degree grade is associated with a higher rate of PhD completion  6. The relationship between PhD success/dropout and change in university is significant, suggesting that pursuing a PhD at the same university leads to a higher success rate.  7. There is a marginally significant relationship between PhD success/dropout and field change. This suggests that pursuing a PhD in the same field could lead to a higher success rate.  8. The relationship between PhD success/dropout and field of research is significant: the field of research has an effect on the rate of PhD completion. Specifically, PhD students in science and technology are more likely to complete their PhD than students in other disciplines.  9. The relationship between PhD success/dropout and funding is significant: PhD students with no funding or unknown funding have the lowest rate of doctoral degree completion.  10. These results show that four factors (marital status, master's degree, field of research, and funding) are directly associated with the attrition rate when all factors are considered together in the same model.  11. The majority of doctoral students drop out in the first two years of their doctoral studies. | No instrument reported (Data bases). | 1. Four factors (marital status, master's degree, field of research, and funding) are directly associated with the attrition rate when all factors are considered together in the same model. 2. Factors such as marital status and gender, interact reciprocally. 3. They found that an accumulation of risk factors leads to a massive increase in attrition rates. |
